# Aspirin: The Mechanism of Action Revisited in the Context of Pregnancy Complications

**DOI:** 10.3389/fimmu.2017.00261

**Published:** 2017-03-15

**Authors:** Angela P. Cadavid

**Affiliations:** ^1^Reproduction Group, Department of Microbiology and Parasitology, School of Medicine, University of Antioquia, Medellín, Colombia

**Keywords:** anti-inflammatory, aspirin-triggered lipoxins, pro-resolving lipid-derived mediators, obstetric antiphospholipid syndrome, preeclampsia, pregnancy complications

## Abstract

Aspirin is one of the most frequently used and cheapest drugs in medicine. It belongs to the non-steroidal anti-inflammatory drugs with a wide range of pharmacological activities, including analgesic, antipyretic, and antiplatelet properties. Currently, it is accepted to prescribe a low dose of aspirin to pregnant women who are at high risk of preeclampsia (PE) because it reduces the onset of this complication. Another pregnancy alteration in which a low dose of aspirin is recommended is the obstetric antiphospholipid syndrome (APS). The most recognized mechanism of action of aspirin is to inhibit the synthesis of prostaglandins but this by itself does not explain the repertoire of anti-inflammatory effects of aspirin. Later, another mechanism was described: the induction of the production of aspirin-triggered lipoxins (ATLs) from arachidonic acid by acetylation of the enzyme cyclooxygenase-2. The availability of a stable analog of ATL has stimulated investigations on the use of this analog and it has been found that, similar to endogenously produced lipoxins, ATL resolves inflammation and acts as antioxidant and immunomodulator. If we consider that in PE and in the obstetric APS, there is an underlying inflammatory process, aspirin might be used based on the induction of ATL. The objective of this review is to revisit the old and new mechanisms of action of aspirin. In particular, it intends to show other potential uses of this drug to prevent certain pregnancy complications in the light of its ability to induce anti-inflammatory and pro-resolving lipid-derived mediators.

## Introduction

Aspirin is the trade name for acetylsalicylic acid coined by the Bayer laboratories. In many countries, it remains a registered trademark of this company, whereas in others aspirin has become the generic name of this substance.

Aspirin in low doses is the single most cost-effective medicine for the prevention of secondary events of thrombosis. Furthermore, low doses of aspirin (LDA) are widely used in the prevention of diverse alterations of gestation such as preeclampsia (PE) and the obstetric antiphospholipid syndrome (APS). Although controversy persists concerning the real efficiency and empirical use of this compound, its prescription is very common in high-risk pregnancies; moreover, its cost is low and it is relatively safe and easily accessible to all (1–7).

As a part of the inflammatory response to an injury, the immune system develops mechanisms of control to this response, through the production of pro-resolving lipid mediators including lipoxins, resolvins, protectins, and maresins. These mediators are produced from arachidonic acid (AA) or from omega-3 polyunsaturated fatty acids (PUFAs), through different molecular mechanisms but that imply transcellular biosynthesis with the participation of different enzymes (8). Interestingly, aspirin induces the production of some pro-resolving lipid-derived mediators very similar to the ones produced endogenously that bind to the same receptor, conferring to aspirin some special properties in the resolution of inflammation (9), in addition to its already known pharmacological effects as analgesic, antipyretic, and antiplatelet drug.

This review aims to revisit the old and new mechanisms of aspirin’s actions and particularly show other possible effects in some complications of pregnancy in which aspirin has been used in an empirical and controversial way.

## A Quick Glance Aspirin-Triggered (AT) the History of Aspirin Discovery

Aspirin is one of the oldest drugs in use, and it is a very representative example of how natural products can be optimized with mild chemical manipulations; its use dates back to 1,500 years B.C., when the Egyptians used crude infusions of myrtle bark for rheumatism and back pain. A thousand years later, Hippocrates prescribed bark and leaves of the willow to relieve fever and pain. In 1763, the Reverend Stone reported a successful treatment of 50 patients in febrile states with willow extract. In 1828, Buchner purified salicin and proposed it as the main component with antipyretic activity of this extract. In 1838, Piria successfully synthesized salicylic acid from salicin. In 1897, Hoffman achieved acetylsalicylic acid as a chemically pure and stable compound; in 1899, its therapeutic properties as an analgesic and anti-inflammatory compound were described and, in 1900, it was introduced into the market in the form of aspirin tablets (10, 11).

For many years, aspirin was widely used as household medicine for the treatment of fever, pain, and inflammation even though its mechanism of action was unknown. It was not until 1971 that the Vane showed that aspirin suppressed the production of some eicosanoids derived from AA such as prostaglandins (12). Later studies demonstrated that the acetylation of platelet cyclooxygenase (COX) by aspirin inhibits thromboxane formation and explains its antithrombotic effects (13).

As of 1979, reports of different actions of aspirin have been flourishing and include its use in the prevention of colon cancer (14), cardiovascular diseases such as myocardial infarction, strokes, and atherothrombotic events (15, 16), as well as the report that regular intake of aspirin during pregnancy reduces the risk of PE (17). One of the discoveries that interests us in the context of this review is the detection in 1989 by Claria and Serhan, of the generation of aspirin-triggered lipoxins (ATLs) from AA, by the interaction of acetylated COX-2 with the 5-lipoxygenase of white cells (18).

## Mechanisms of Action of Aspirin

**Figure 1:**
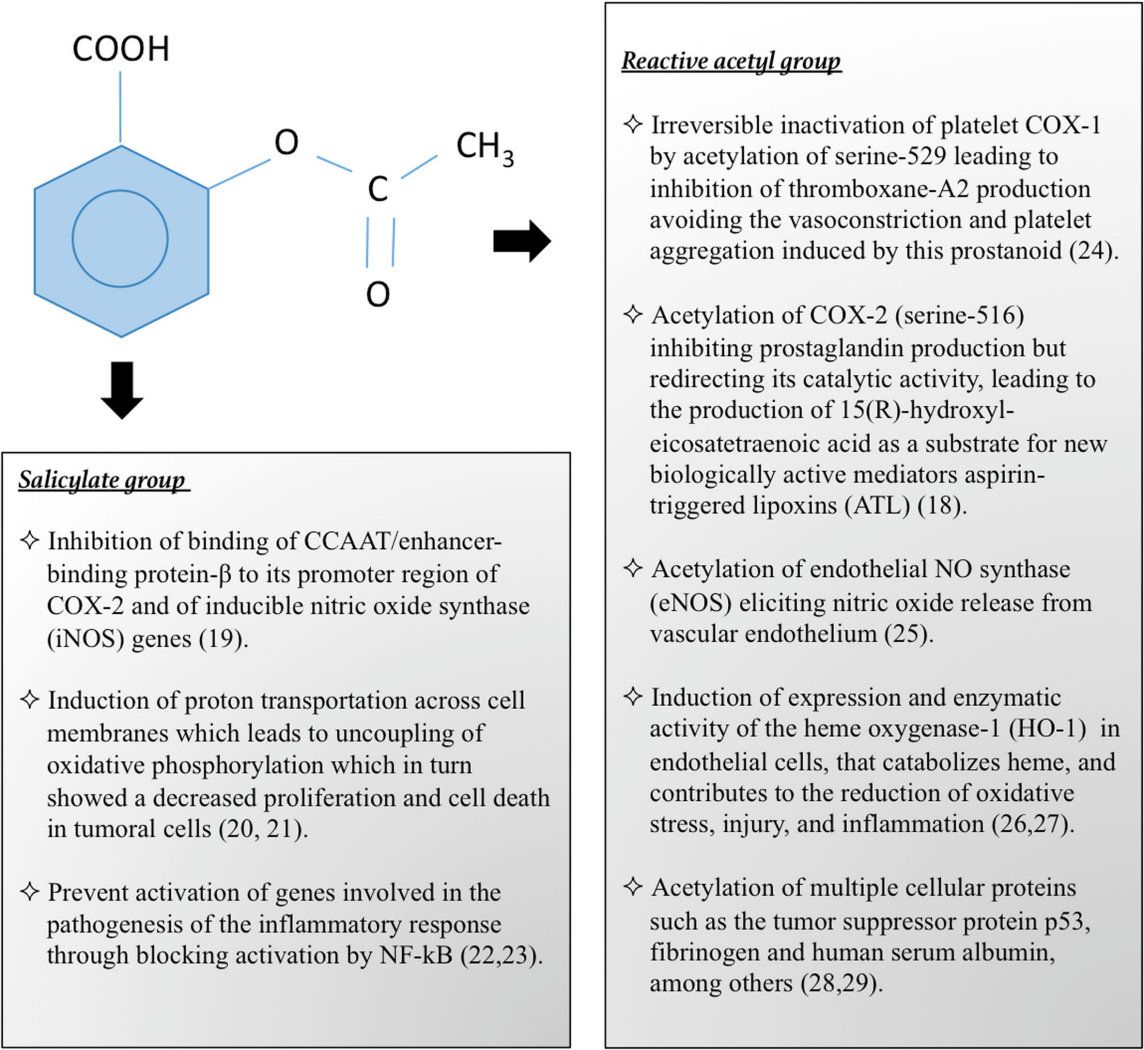
**Pharmacological and biological actions of aspirin by its salicylate and reactive acetyl group**.

Aspirin is a prototype of non-steroidal anti-inflammatory drugs (NSAIDs), and member of the family of salicylates that have in common salicylic acid as the active agent. Salicylic acid is composed of a benzene ring and two radicals, one hydroxyl and one carboxyl. In the acetylsalicylic acid or aspirin, the hydroxyl group salicylate is transformed into an acetyl group by esterification. The pharmacological properties of aspirin are similar to those of salicylates, but also to the biological actions attributed to salicylate itself, and it has other independent effects due to its reactive acetate group (11). Both components, salicylate and acetate groups, are biologically active and act independently of each other at different sites. A summary of the pharmacological actions of these components of aspirin are summarized in Figure 1 (18–29).

**Figure 2:**
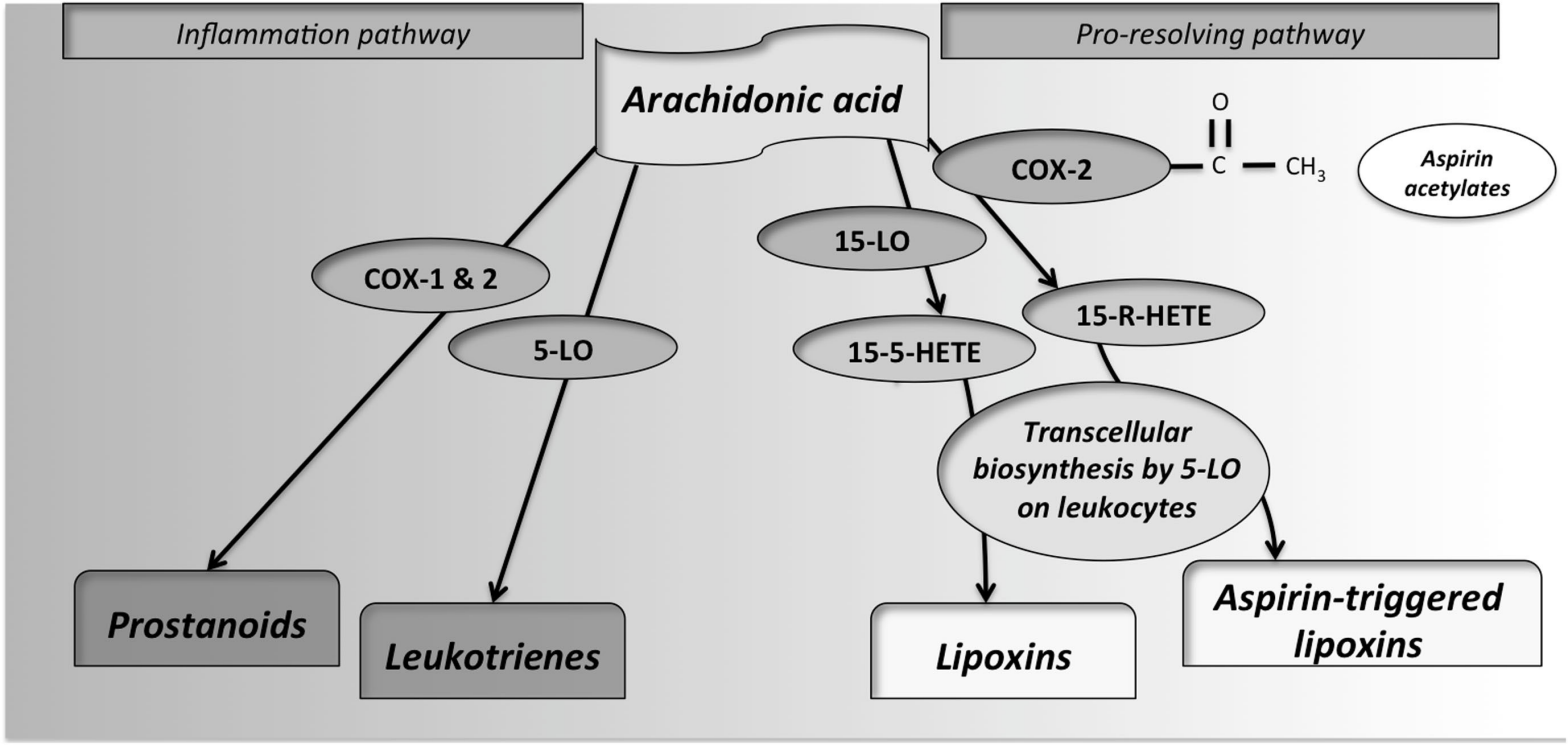
**Synthesis of pro-inflammatory and pro-resolving lipid mediators from arachidonic acid (AA)**. By the action of cyclooxygenases-1 and -2, the prostanoids prostacyclins, prostaglandins and thromboxanes, are produced. These enzymes are inhibited by non-steroidal anti-inflammatory drugs, including aspirin. If AA interacts with 5-lypoxigenase (5-LO), leukotrienes, also important mediators of inflammation, are produced. In the control of inflammatory response, the metabolite 15(S)-hydroxy-eicosatetraenoic acid (15S-HETE) is produced from LO from different cellular sources. This metabolite, through interaction with 5-LO in leukocytes by transcellular biosynthesis, produces some lipid mediators so-called lipoxins. Additionally, as an exclusive property of aspirin, by its reactive acetate group, aspirin can acetylate the active site of cyclooxygenase (COX)-2. This interaction inhibits its catalytic activity as a COX but redirects it, leading to the production of 15R-HETE from AA. 15R-HETE is then also converted through transcellular biosynthesis, by white-cell 5-LO, into aspirin-triggered lipoxins.

LDA (e.g., 75–100 mg/day) are sufficient to irreversibly acetylate serine 530 of COX-1, inhibiting platelet generation of thromboxane-A2, resulting in an antithrombotic effect. Intermediate doses of aspirin (650 mg to 4 g/day) inhibit COX-1 and COX-2 (30). Additionally, aspirin can induce the production of ATL (18). This lipid mediator exerts its actions by binding to a G-protein-coupled receptor, named ALXR (9). A simple scheme of the metabolic pathways of AA is shown in Figure 2.

## Use of Aspirin in Prevention of PE

Preeclampsia is a multisystem disorder defined by persistent hypertension during pregnancy or postpartum period that may be associated with proteinuria, thrombocytopenia, impaired liver function, progressive renal insufficiency, pulmonary edema, or cerebral disturbances (31). It is generally accepted that PE originates from the placenta, since poor trophoblast invasion and remodeling of spiral arteries occur, leading to reduced utero-placental arterial flow and episodes of hypoxia/reperfusion (32). These abnormalities in the perfusion of placenta generate reactive oxygen species that, after a while, result in the release of cytokines, lipid peroxides, and syncytiotrophoblast microfragments from the placenta into the maternal circulation (33). Hence, in PE, the increased inflammation, oxidative stress, and endothelial dysfunction are key pathogenic features (34).

Since 1979, when the utility of aspirin intake in pregnancy was reported to prevent PE (17), many reports with controversial results on the efficiency of this drug were reported: two multicenter studies found a slight benefit of aspirin in preventing PE (35, 36). Other randomized placebo-controlled trials suggest that LDA did not reduce the rate of PE [relative risk (RR), 0.7, 95% confidence interval (CI), 0.3–1.7] (37). On the other hand of the controversy, other reports in the literature showed results in favor of the beneficial effects of LDA to prevent PE: in a systematic review of the literature of 46 trials involving 32,891 patients, a moderate benefit of aspirin was found in preventing PE; LDA reduced the risk of PE by 17% (RR, 0.83, 95% CI, 0.77–0.89). These results were statistically significant independent of whether patients had moderate or high risk of PE, or whether they were included in a placebo-controlled trial (1). In a recent meta-analysis, it was shown that LDA used before the 16th week of pregnancy reduces the risk of PE (RR, 0.57; 95% CI, 0.43–0.75; *p* < 0.001) (3). However, other authors did not find differences in the beneficial effect of aspirin whether treatment was started before or after 16 weeks of gestation (38).

Regarding the risks of using LDA during pregnancy, most studies have found no association between its use and complications in the mother or fetus, whether used in the first or third trimester. These studies show the lack of association of the use of such treatment with congenital anomalies, neonatal intraventricular hemorrhage, premature closure of the ductus arteriosus, maternal postpartum bleeding, or placental abruption (1, 39, 40). An adverse event such as vaginal bleeding not associated with gestational loss was described (41). Other maternal factors such as allergy or resistance to aspirin, in addition to gastric intolerance, could counterindicate the use of LDA in pregnancy (42).

Despite these controversial results, the preventive use of LDA in women at high risk for PE such as medical history of previous severe-PE, diabetes, chronic hypertension, renal disease, or autoimmune disease seems to be accepted (1, 39, 40). However, the controversy regarding the use of LDA persists in low-risk women. Currently, a panel of diagnostic tests exists to determine PE risk that includes uterine artery Doppler pulsatility index, and some placental biomarkers such as pregnancy-associated plasma protein A and placental growth factors (PLGF) (42). However, in developing countries, it is costly to have access to such tests and in that sense, it would be beneficial to recommend the use of LDA in women in whom PE risk is suspected.

## Use of Aspirin in Obstetric APS

Antiphospholipid syndrome is an autoimmune disorder characterized by the persistent presence of antiphospholipid (aPL) antibodies and clinical manifestations of vascular thrombosis or obstetrical complications, and also both aspects of the syndrome. Clinical criteria for obstetric APS include at least one of the following pathologies: early or late gestational loss, intrauterine growth restriction, placental insufficiency, or PE (43).

The mechanisms of injury of aPL involve activation of the endothelium, platelets, and monocytes, and complement activation and inhibition of anticoagulant proteins, leading to the phenomena of inflammation and thrombosis. It has been demonstrated that aPL-associated obstetric complications are induced mainly by an inflammatory process and placental insufficiency rather than by thrombotic events at the maternal–fetal interface (44–49). aPL interferes with the migration, proliferation, and differentiation of trophoblast cells and decreases the production of placental human chorionic gonadotropin (hCG) (47, 50–58). Furthermore, aPL impairs interaction between the invading trophoblast and the endothelium of the uterine spiral arteries, which is a key process where spiral arteries are transformed into high-capacity low-resistance vessels to supply the growing nutritional demands of the fetus and the placenta (59).

Low doses of aspirin, alone or combined with low molecular heparin, is one of the preferred treatments in pregnant women with obstetric APS (4, 5, 60). Although a meta-analysis published in 2005 concluded that the combination of LDA plus unfractioned heparin had up to a 54% chance of reducing fetal loss (61), a subsequent randomized controlled-clinical trial did not find significant differences between the observed outcome for APS patients treated with aspirin alone in comparison with combined therapy of aspirin plus low molecular weight heparin (79.1 and 77.8% of live births, respectively) (60). Because these protocols fail in about 20% of pregnant APS women, additional therapies have been proposed to add to conventional therapies (62, 63).

Low doses of aspirin reduced embryo resorption in a model of experimental APS induced in pregnant mice (64) and restored placental hCG secretion abolished by the effect of aPL (47). Over a period of 25 years, in our Reproduction Group of the University of Antioquia, LDA has been used alone or combined with other therapies to prevent recurrent spontaneous abortion. The other therapies include progesterone, heparin, folic acid, or lymphocyte immunotherapy. In a group of 111 women with a history of three or more abortions, who were treated with aspirin during their pregnancy, a coadjuvant effect of the treatment including aspirin was observed: if the patients who received some treatment were compared with patients non-treated at all, the odds ratio (OR) was 0.33 (0.13–0.81, *p* = 0.01); if the treatment including aspirin was compared with no treatment, the OR was 0.13 (0.04–0.43, *p* < 0.001) (65). Additionally, a prospective study of “single therapy” with aspirin/heparin in patients with recurrent spontaneous abortion was carried out. These patients displayed autoimmune and alloimmune alterations, but lymphocyte immunotherapy was not administered to them; the gestational success in this group of patients was of 90.9% (10/11) versus 75.0% (6/8) in the concurrent group receiving both therapies (66). These results are empirical and lack the rigor of controlled-clinical trials, but encouraged us to continue exploring the use of LDA as a simple therapy in patients with recurrent pregnancy loss, associated or not with obstetric APS.

## Clinical and Experimental Effects of ATL

Aspirin-triggered lipoxin promotes the resolution of inflammation and acts as antioxidant and immunomodulator. It also blocks the generation of reactive oxygen species in endothelial cells; it is a potent anti-inflammatory factor, inhibiting leukocyte–endothelial interaction and cell chemiotaxis of neutrophils while promoting monocyte chemiotaxis and non-phlogistic phagocytosis of apoptotic neutrophils by macrophages; it inhibits NF-κB activation, and TNF-α secretion in activated T cells (67–69). The effect of ATL has been used successfully in a wide range of murine disease models such as experimental asthma, trimellitic anhydride-induced delayed type of hypersensitivity reaction, chronic airway inflammation, and infection associated with cystic fibrosis. Additionally, ATL inhibits proliferation and angiogenesis in proliferative states such as chronic inflammation, ischemic diseases, and cancer (67, 70–72). These results open a range of potential therapeutic uses of ATL in a variety of inflammatory diseases.

Some authors have shown that the anti-inflammatory activity of aspirin is due to the production of nitric oxide (NO) and that this effect is mediated by the ATL-induced NO synthesis through constitutive and inducible NO synthases (eNOS and iNOS, respectively) (25, 73). Meanwhile other authors argue that in the aspirin-mediated NO production the heme oxygenase-1 protein (HO-1) is involved, suggesting that more than one signaling pathway may be implicated. HO-1 is the inducible enzyme that catabolizes heme, leading to the generation of bilirubin, carbon monoxide, and iron. These molecules have antioxidant, antiapoptotic, and cytoprotective properties (74, 75).

Although the usefulness of the ATL in the field of reproduction has not been studied extensively, some reports suggest the possibilities for its use in this context. An increase of ALXR expression in the human endometrium during the menstrual cycle and in decidua during the first trimester of pregnancy was observed (76). In our group, we evaluated the effect of ATL on the inflammatory and oxidative response induced by plasma from preeclamptic women on endothelial cells (human umbilical venous endothelial cells). First, increased amounts of antiangiogenic factors (sFlt-1), pro-inflammatory (TNF) mediators, and products of lipid peroxidation (TBARS and 8-isoprostane) in preeclamptic plasma were observed. Besides, leukocyte adhesion to endothelial cells was evaluated and both preeclamptic plasma and exogenous TBARS, and 8-isoprostane increased neutrophil adhesion to these cells, and this inflammatory response was reduced when neutrophils were incubated with ATL prior to coculture with endothelial cells (77). On the other hand, we assessed the modulatory effects of ATL over some aPL-altered trophoblast functions: the monoclonal anti-β_2_GPI antibodies ID2 and IIC5 significantly reduced spontaneous trophoblast cell migration and also disrupted the trophoblast–endothelial cell interactions evaluated by a three-dimensional *in vitro* system of vascular remodeling. Both, migration and stability of the cocultures, were restored with simultaneous incubation with ATL. Similar results were obtained with serum samples from aPL-positive patients either with pregnancy morbidity alone (PM serum) or pregnancy morbidity plus vascular thrombosis (PM/VT) but ATL only restored aPL-altered trophoblast functions in the PM group, which could be an indication that the additional use of heparin is required in patients with PM/VT. An anti-inflammatory effect of ATL could not be demonstrated in these assays since ATL treatment did not resolve the aPL-induced pro-inflammatory and antiangiogenic responses of trophoblasts evaluated in terms of trophoblast secretion of the pro-inflammatory chemokine IL-8, the proangiogenic factor placental growth factor (PLGF) or the antiangiogenic factor soluble endoglin (59). As a whole, these findings the possibility of using ATL as an adjuvant therapy for women with PE or obstetric APS.

## Aspirin-Induced Lipid Mediators Derived from Omega-3 PUFAs: Do They have Some Potential Use in Pregnancy Complications?

Other lipid mediators derived from omega-3 PUFAs, eicosapentaenoic acid (EPA) and docosahexaenoic acid (DHA), have been described more recently. EPA-derived mediators include resolvin E1 (RvE1) and RvE2; the DHA-derived mediators described are D-series resolvins, protectin D1, and maresins. Aspirin can induce the formation of RvE1 and the AT D-series resolvins, in a similar way to those of ATL (9). These mediators have potent pro-resolving inflammation activities (8). There are very few reports of the effect of endogenous lipid mediators or AT mediators derived from omega-3 PUFAs in pregnancy (78), but it has been reported that the dietary intake of these fatty acids increases resolvin and protectin levels in the rat placenta (79), and it has been proposed that omega-3 supplementation prevents preterm birth in humans (80).

Theoretically, the combination of omega-3 and LDA could have a synergistic effect in controlling inflammation. Some studies have found this effect in different scenarios: in TLR-7-activated microglia cells (81), in the treatment of three patients with progressive IgA nephropathy (82), and in the decrease of atherosclerosis in apoE-null mice (83). On the other hand, other authors, in a group of healthy volunteers, did not find any effect of aspirin on the production of pro-resolving lipid mediators (84), nor that the presence of aspirin had any additional effect to that of the omega-3 PUFA in decreasing markers of inflammation (85). The beneficial effect it could have by the combination of omega-3 PUFAs and LDA in preventing pregnancy complications such as PE, based on the production of AT-resolvins requires further studies.

## Concluding Remarks

Aspirin, and particularly LDA, has a therapeutical potential beyond its already known effects in the prevention of several diseases such as myocardial infarction, strokes, atherothrombotic events, PE, and colon cancer. Besides the pharmacological effects that it shares with other NSAIDs, aspirin can induce other lipid-derived mediators with potent anti-inflammatory actions, and stimulation of the resolution of inflammation places aspirin in a privileged position in the therapeutic arsenal. In the context of prevention of some alterations of pregnancy, the prescription of drugs must be particularly careful to minimize the risk in both mother and fetus and even though aspirin is not exempt of risks, the risk–benefit balance is directed in favor of the beneficial effects. The use of LDA to prevent pregnancy complications such as PE and obstetric APS has been based on the restauration of the prostacyclin/thromboxane-A2 balance to the dominance of the former. This action is due to aspirin’s property to inhibit COX: platelets do not synthetize new protein, but endothelial cells do. However, in the light of newly identified mechanisms of action of aspirin, other immunomodulatory, anti-inflammatory, and antioxidant effects might be explored. The proposed challenge is a deep study of the molecular mechanisms implied in the effects of aspirin and of AT mediators to propose a more rational use of it based on the selection of patients who could benefit from aspirin, when the treatment should begin, and the dose that should be used.

## Author Contributions

APC wrote the manuscript and approved it for publication.

## Conflict of Interest Statement

The author declares that the research was conducted in the absence of any commercial or financial relationships that could be construed as a potential conflict of interest.
